# Supplementary material for: Shape of the first mitotic spindles impacts multinucleation in human embryos
Source: Nat Commun. 2024 Jun 25;15:5381. doi: 10.1038/s41467-024-49815-8 (PMC11199590; doi:10.1038/s41467-024-49815-8)
Supplement: Supplementary file 1 — Supplementary Information [file 41467_2024_49815_MOESM1_ESM.pdf]

Supplementary information

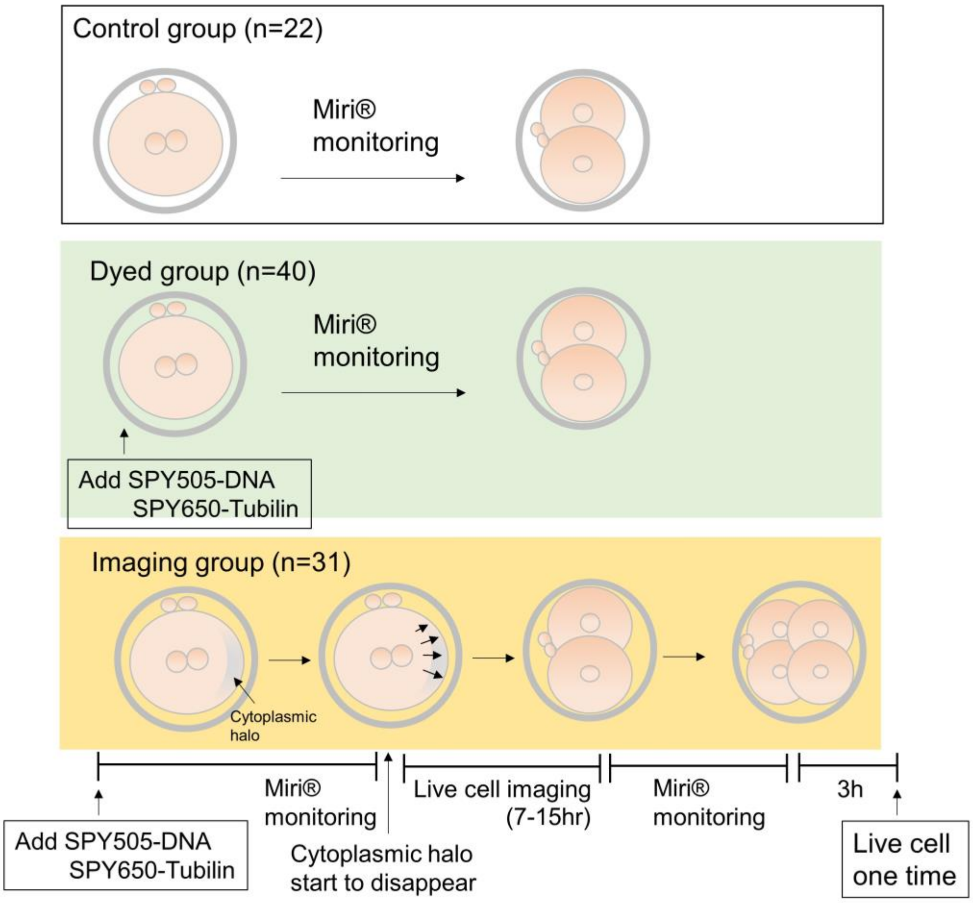

Supplementary Figure 1. Embryos used in this study. Schematic outlining steps for embryos in control, dyed, and imaged groups.

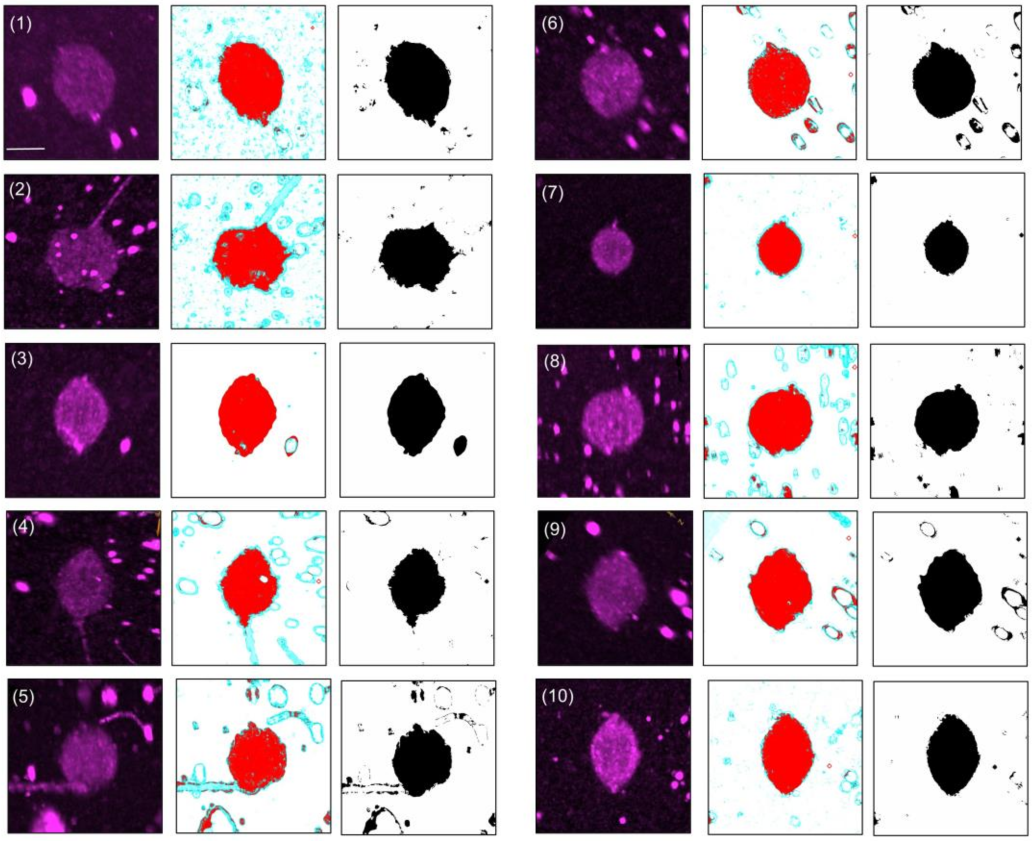

Supplementary Figures 2. Spindle images for embryos 1 – 10 analyzed in this study.

The numbers in the images represent the embryo numbers, which correspond to Supplementary Table 1. The image at the end of metaphase from an angle shows chromosomes aligned linearly and where the spindle appears to have the maximum area (left). Image processing using Ilastik. The objects were classified using an image recognition algorithm, and the shape of the spindle was extracted as a red area. The background and parts of the sperm tail were excluded as they were blue (middle). The binarized image (middle) was created by extracting only the red area (right). Scale bar: 10 µm

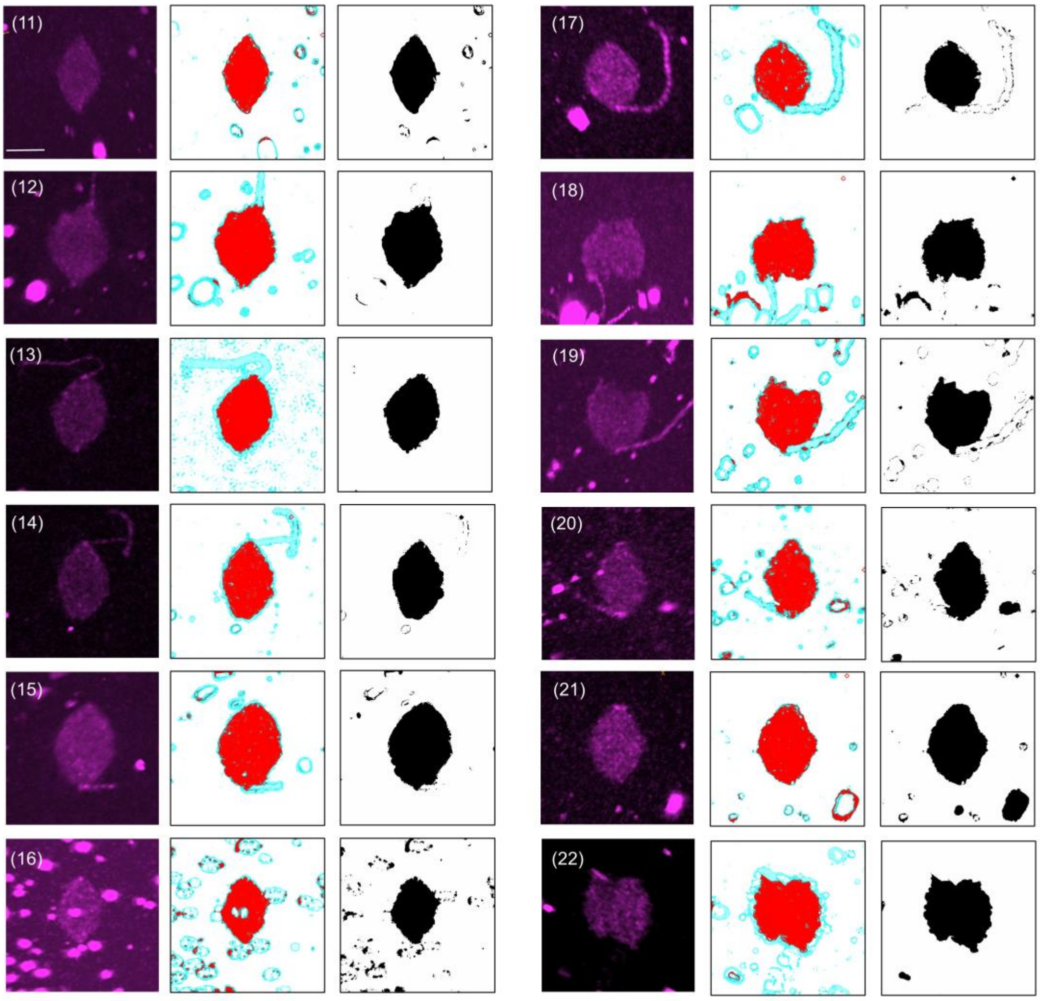

Supplementary Figures 3 Spindle images for embryos 11 – 22 analyzed in this study.

The numbers in the images represent the embryo numbers, which correspond to Supplementary Table 1. The image at the end of metaphase from an angle shows chromosomes aligned linearly and where the spindle appears to have the maximum area (left). Image processing using Ilastik. The objects were classified using an image recognition algorithm, and the shape of the spindle was extracted as a red area. The background and parts of the sperm tail were excluded as they were blue (middle). The binarized image (middle) was created by extracting only the red area (right). Scale bar: 10 μm

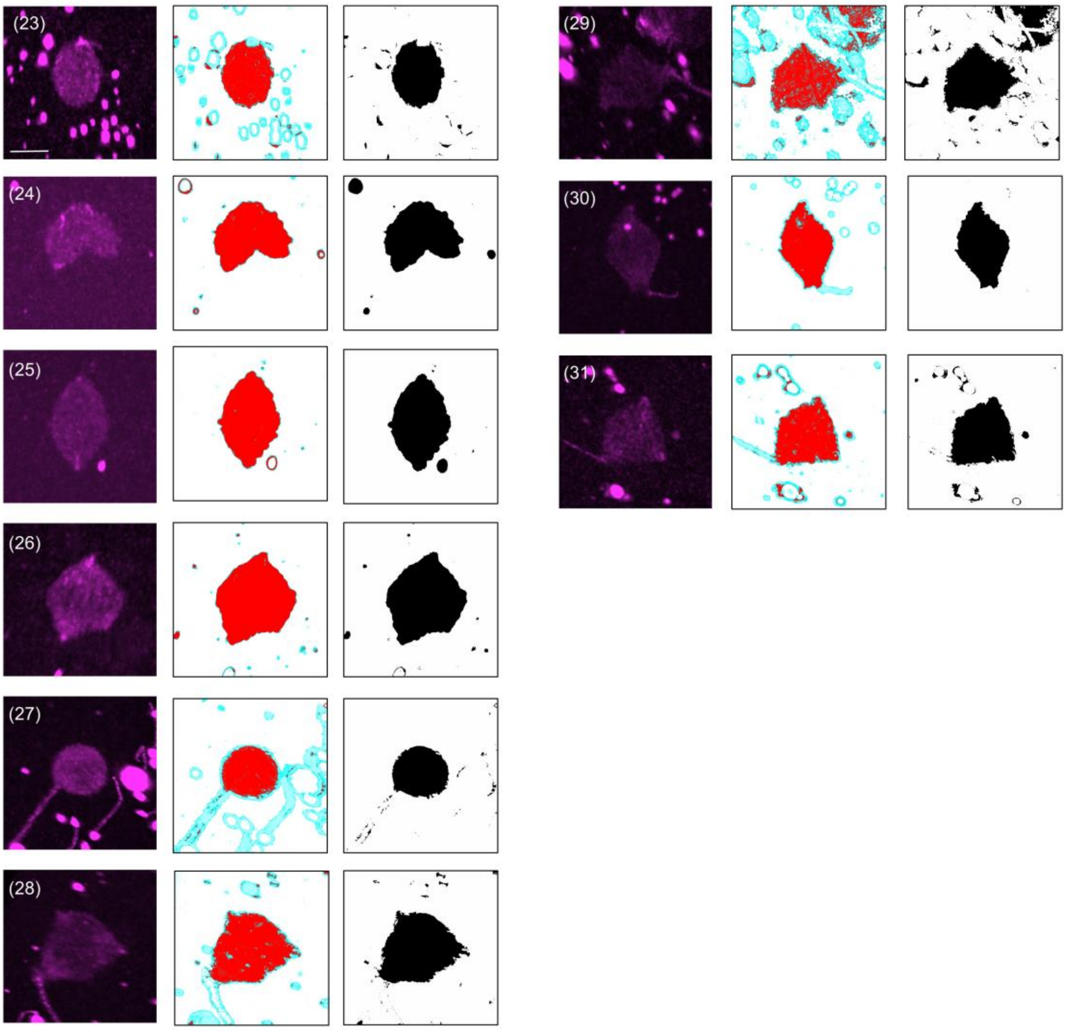

Supplementary Figures 4 Spindle images for embryos 23-32 analyzed in this study. The numbers in the images represent the embryo numbers, which correspond to Supplementary Table 1. The image at the end of metaphase from an angle shows chromosomes aligned linearly and where the spindle appears to have the maximum area (left). Image processing using Ilastik. The objects were classified using an image recognition algorithm, and the shape of the spindle was extracted as a red area. The background and parts of the sperm tail were excluded as they were blue (middle). The binarized image (middle) was created by extracting only the red area (right). Scale bar: 10 μm

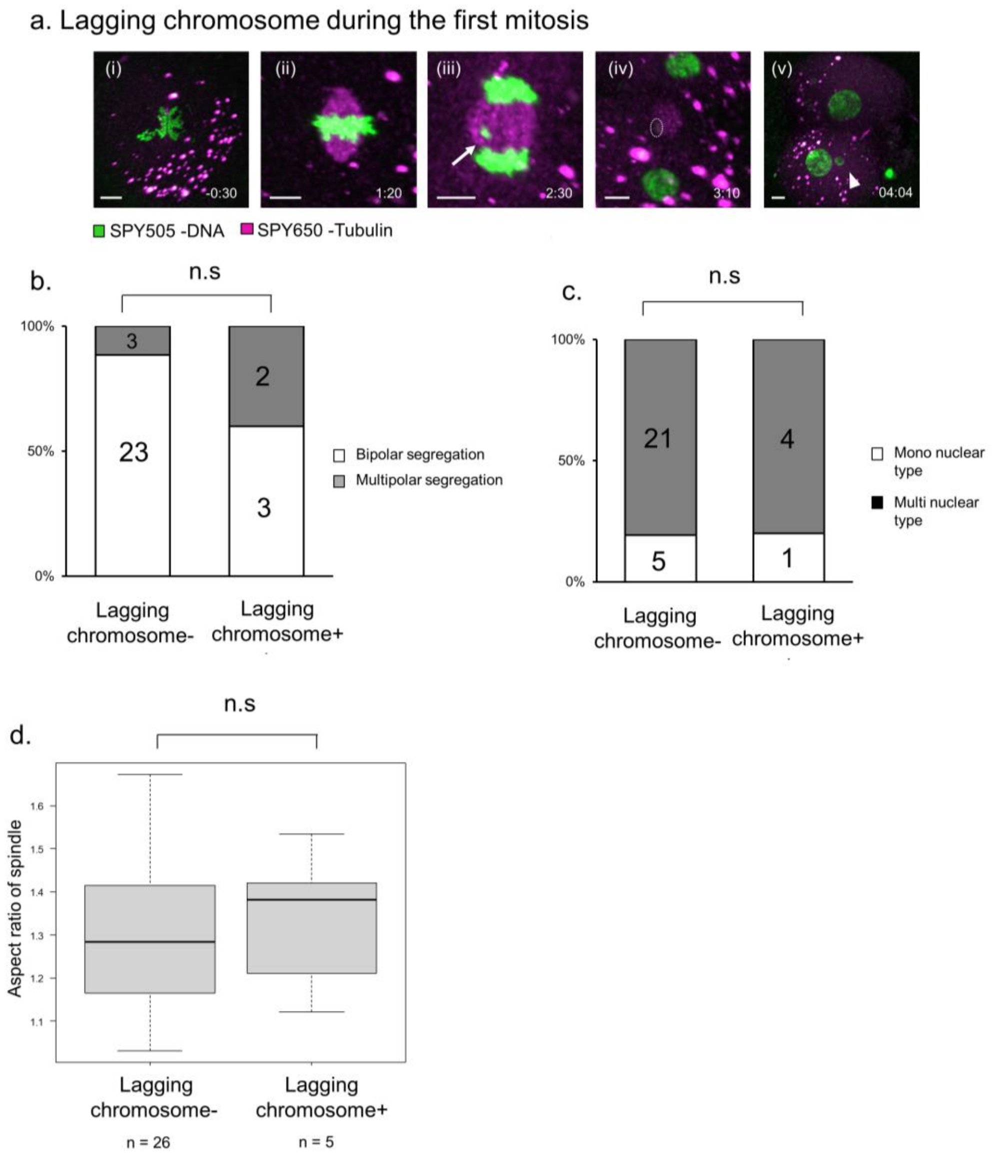

Supplementary Figures 5

Lagging chromosomes occur independent shape of spindle in the first mitosis.

a, Time-lapse live imaging of representative confocal microscopic images of a human embryo exhibiting a lagging chromosome. The white arrow (iii) and white circle (iv) indicate a lagging chromosome. The white arrowhead indicates micronucleus. Scale bar, (i)15 μm, (ii) - (v) 10 μm.

b, Quantification of anaphase errors in all imaged embryos arising a lagging chromosome. A two-sided Fisher's exact test was performed. p-value = 0.173. Source data are provided as a Source Data file. **c**, Quantification of poles

focusing or defocusing in all imaged embryos arising a lagging chromosome. A two-sided Fisher's exact test was performed. p-value = 1. Source data are provided as a Source Data file. **d,** Quantification of spindle AR for all image embryos arising a lagging chromosome during the first mitosis. A two-tailed Mann-Whitney U-test was performed. p-value = 0.584. Source data are provided as a Source Data file.

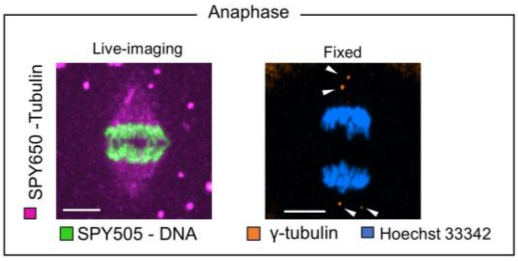

Supplementary Figures 6

Centrosomes in anaphase during the first mitosis in human embryo.

Live-imaging and immunofluorescence staining of ***γ***-tubulin and chromosomes (Hoechst33342) in human zygotes. Scale bar, 10 μm. White arrowheads indicate ***γ***-tubulin positive MTOC.

	Bipolar focused spindle (n=15)	Bipolar defocused spindle (n=10)	Multipolar spindle (n=5)	P-value
Prophase (min)	20.00 [9.5]	29.00 [10.0]	20.00 [5.3]	0.415
Metaphase (min)	99.00 [27.5]	110.00 [21.0]	100.00 [20.0]	0.382
Anaphase (min)	49.50 [10.0]	50.00 [10]	40.00 [0.0]	0.494
Duration of spindle formation (min)	30.00 [29.25, 38.00]	30.00 [30.00, 45.00]	29.50 [26.75, 47.50]	0.841
Duration when spindles start to form until it elongates (min)	129.00 [119.75, 146.00]	150.00 [139.75, 179.25]	140.00 [137.25, 142.50]	0.206

Values are presented as median +- IQR

Supplementary Table 1

Median durations of each mitotic phase and spindle phase by spindle shape type. Square brackets indicate IQR. A two-tailed Mann-Whitney U-test was performed. Source data are provided as a Source Data file.

	Mono nuclear type (n=7)	Multi nuclear type (n=25)	P-value
Prophase (min)	24.00 [8.8]	20.00 [10.0]	0.546
Metaphase (min)	99.50 [8.3]	100.00 [40.0]	0.641
Anaphase (min)	49.00 [10.0]	45.00 [10.0]	0.844
Duration of spindle formation (min)	30.00 [7.3]	30.00 [10.8]	0.896
Duration when spindles start to form until it elongates (min)	134.50 [18.0]	140.00 [21.8]	0.347

Values are presented as median +- IQR

Supplementary Table 2

Median durations of each mitotic phase and spindle phase by nuclear type. Square brackets indicate IQR. A two-tailed Mann-Whitney U-test was performed. Source data are provided as a Source Data file.

embryo No.	PNBD-furrow ingression(hrs)	segregation type	aspect ratio of spidle	focued or defocused pole	defocued pole type	defocused pole side	nucleation status at 2 cell stage
1	2.6	bipolar	1.36	defocused	unilateral	no sperm tail	multi
2	2.2	bipolar	1.18	defocused	bilateral		multi
3	2.7	bipolar	1.42	focused			multi
4	2.8	bipolar	1.34	focused			multi
5	2	bipolar	1.16	defocused	bilateral		multi
6		bipolar	1.17	defocused	unilateral	no sperm tail	multi
7		bipolar	1.21	focused			multi
8	2	bipolar	1.09	defocused	bilateral		multi
9	2	bipolar	1.29	focused			multi
10	2.5	bipolar	1.53	focused			mono
11	2.6	bipolar	1.67	focused			mono
12	2.5	bipolar	1.33	defocused	unilateral	sperm tail	multi
13	2.3	bipolar	1.44	focused			multi
14	2.7	bipolar	1.53	focused			mono
15	2.5	bipolar	1.38	focused			multi
16	2.4	bipolar	1.42	focused			mono
17	2.3	bipolar	1.20	focused			multi
18	3.7	bipolar	1.05	defocused	bilateral		multi
19		bipolar	1.03	defocused	unilateral	no sperm tail	multi
20		bipolar	1.54	focused			multi
21	2.3	bipolar	1.39	focused			multi
22	2.5	bipolar	1.17	defocused	bilateral		multi
23	2.8	bipolar	1.29	focused			mono
24	2.5	multipolar	1.28				multi
25	2.7	bipolar	1.58	focused			mono
26	2.8	bipolar	1.15	defocused	unilateral	no sperm tail	multi
27	2.9	multipolar	1.12				multi
28	2.8	multipolar	1.21				multi
29	3.5	multipolar	1.20				multi
30	3.3	bipolar	1.65	defocused	unilateral	sperm tail	multi
31	2.5	multipolar	1.11				multi

Supplementary Table 3. Spindle detailed information of all embryos.

All embryos were imaged during the first mitosis. Source data are provided as a Source Data file.
