# Supplementary material for: Shape of the first mitotic spindles impacts multinucleation in human embryos
Source: Nat Commun. 2024 Jun 25;15:5381. doi: 10.1038/s41467-024-49815-8 (PMC11199590; doi:10.1038/s41467-024-49815-8)
Supplement: Supplementary file 3 — Description of Additional Supplementary Files [file 41467_2024_49815_MOESM3_ESM.pdf]

Description of Additional Supplementary Files

File Name: Supplementary Movie 1
Description: Time-lapse live imaging of representative confocal microscopic movie of human embryos undergoing bipolar segregation using fluorescent labeling.

File Name: Supplementary Movie 2
Description: Time-lapse live imaging of representative confocal microscopy movie of human embryos undergoing multipolar segregation

File Name: Supplementary Movie 3
Description: Time-lapse live imaging of representative confocal microscopic movie of human embryos exhibiting a high-AR (1.67) spindle

File Name: Supplementary Movie 4
Description: Time-lapse live imaging of representative confocal microscopic movie of human embryos exhibiting a low-AR (1.18) spindle.

File Name: Supplementary Movie 5
Description: Time-lapse live imaging of representative confocal microscopic movie of a human embryo exhibiting a spindle with a unilateral defocused pole.

File Name: Supplementary Movie 6
Description: Time-lapse live imaging of representative confocal microscopic movie of a human embryo that has a spindle with a bilateral defocused pole.

File Name: Supplementary Movie 7
Description: Time-lapse live imaging of representative confocal microscopic movie of a human embryo exhibiting a lagging chromosome from prophase to telophase

File Name: Supplementary Movie 8
Description: Time-lapse live imaging of representative confocal microscopic images of a human embryo exhibiting a lagging chromosome after cytokinesis.

File Name: Supplementary Movie 9
Description: Time-lapse live imaging of representative confocal microscopy movie of the second mitosis in human embryos with multiple nuclei at the 2-cell stage.
