# Supplementary material for: Shape of the first mitotic spindles impacts multinucleation in human embryos
Source: Nat Commun. 2024 Jun 25;15:5381. doi: 10.1038/s41467-024-49815-8 (PMC11199590; doi:10.1038/s41467-024-49815-8)
Supplement: Supplementary file 13 — Reporting Summary [file 41467_2024_49815_MOESM13_ESM.pdf]

nature portfolio

Corresponding author(s): Yuki Ono

Last updated by author(s): June 1, 2024

Reporting Summary

Nature Portfolio wishes to improve the reproducibility of the work that we publish. This form provides structure for consistency and transparency in reporting. For further information on Nature Portfolio policies, see our Editorial Policies and the Editorial Policy Checklist.

Statistics

For all statistical analyses, confirm that the following items are present in the figure legend, table legend, main text, or Methods section.

n/a	Confirmed	
☐	☒	The exact sample size (*n*) for each experimental group/condition, given as a discrete number and unit of measurement
☐	☒	A statement on whether measurements were taken from distinct samples or whether the same sample was measured repeatedly
☐	☒	The statistical test(s) used AND whether they are one- or two-sided<br>*Only common tests should be described solely by name; describe more complex techniques in the Methods section.*
☒	☐	A description of all covariates tested
☐	☒	A description of any assumptions or corrections, such as tests of normality and adjustment for multiple comparisons
☐	☒	A full description of the statistical parameters including central tendency (e.g. means) or other basic estimates (e.g. regression coefficient) AND variation (e.g. standard deviation) or associated estimates of uncertainty (e.g. confidence intervals)
☐	☒	For null hypothesis testing, the test statistic (e.g. F, t, r) with confidence intervals, effect sizes, degrees of freedom and P value noted<br>*Give P values as exact values whenever suitable.*
☒	☐	For Bayesian analysis, information on the choice of priors and Markov chain Monte Carlo settings
☒	☐	For hierarchical and complex designs, identification of the appropriate level for tests and full reporting of outcomes
☒	☐	Estimates of effect sizes (e.g. Cohen's *d*, Pearson's *r*), indicating how they were calculated

Our web collection on statistics for biologists contains articles on many of the points above.

Software and code

Policy information about availability of computer code

Data collection	Time-lapse imaging of live samples were both performed using ZEN Blue imaging software (Zeiss), on a LSM 980(Zeiss).
Data analysis	All image analyses were performed using Imaris 10.0 (Oxford Instruments), Fiji, and Ilastik . Statistical analyses were performed using EZR, a modified version of R.

For manuscripts utilizing custom algorithms or software that are central to the research but not yet described in published literature, software must be made available to editors and reviewers. We strongly encourage code deposition in a community repository (e.g. GitHub). See the Nature Portfolio guidelines for submitting code & software for further information.

Data

Policy information about availability of data

All manuscripts must include a data availability statement. This statement should provide the following information, where applicable:

- Accession codes, unique identifiers, or web links for publicly available datasets
- A description of any restrictions on data availability
- For clinical datasets or third party data, please ensure that the statement adheres to our policy

The primary confocal microscopy data were not uploaded to a data repository because of their large size but are available from the authors upon request.

Research involving human participants, their data, or biological material

Policy information about studies with human participants or human data. See also policy information about sex, gender (identity/presentation), and sexual orientation and race, ethnicity and racism.

Reporting on sex and gender	Human embryos were used.
Reporting on race, ethnicity, or other socially relevant groupings	Human embryos were used.
Population characteristics	Embryo donors were patients treated in a fertility clinic. Female donors were between the ages of 27 to 39 years old.
Recruitment	Participation in this study was entirely voluntary and no financial inducements were given for embryo donation. Embryo donors were informed that their donated surplus embryos would be used to study the developmental mechanisms of human embryos and that their donation would not affect their IVF cycle. All embryo donors signed informed consent forms stating clearly the goals of the research, potential benefits and risks, and steps taken to ensure that their privacy was met. Conditions of donation: not for profit. the detailed patient information, such as underlying disease and method of ovarian stimulation, was not available. It is possible that the human zygotes used in this study had a bias in patient characteristics.
Ethics oversight	This study was approved by the Ethics Committee of Akita University (approval number: 1090-2), Japan Society of Obstetrics and Gynecology (Registry No. 75), and Asada Ladies Clinic (approval number: 2022-13). We obtained written informed consent from patients who donated human embryos. The experiments were performed in accordance with the relevant regulations on human sperm/ovum/fertilized eggs set forth by the Japan Society of Obstetrics and Gynecology.

Note that full information on the approval of the study protocol must also be provided in the manuscript.

Field-specific reporting

Please select the one below that is the best fit for your research. If you are not sure, read the appropriate sections before making your selection.

☒ Life sciences ☐ Behavioural & social sciences ☐ Ecological, evolutionary & environmental sciences

For a reference copy of the document with all sections, see nature.com/documents/nr-reporting-summary-flat.pdf

Life sciences study design

All studies must disclose on these points even when the disclosure is negative.

Sample size	No statistical test was performed to determine sample size because it was very difficult to obtain enough human two-pronuclear-stage embryos. Sample size was determined based on prior similar research using the two-pronuclear-stage human embryo; Currie, C. E. et al. Nat. Commun. 13, 6755 (2022).
Data exclusions	Embryos excluded where the entire spindle was not imaged because embryos moving.
Replication	All experiments in this study were successfully performed at least 10 times with the same and different stage of human embryos.
Randomization	Embryos were randomly allocated into experimental groups.
Blinding	Successfully developed and imaged embryos have to be selected for subsequent analysis. Therefore, investigators were only blinded for computational analysis following acquisition of imaging data.

Reporting for specific materials, systems and methods

We require information from authors about some types of materials, experimental systems and methods used in many studies. Here, indicate whether each material, system or method listed is relevant to your study. If you are not sure if a list item applies to your research, read the appropriate section before selecting a response.

Materials & experimental systems

n/a	Involved in the study
☐	☒ Antibodies
☒	☐ Eukaryotic cell lines
☒	☐ Palaeontology and archaeology
☒	☐ Animals and other organisms
☒	☐ Clinical data
☒	☐ Dual use research of concern
☒	☐ Plants

Methods

n/a	Involved in the study
☒	☐ ChIP-seq
☒	☐ Flow cytometry
☒	☐ MRI-based neuroimaging

Antibodies

Antibodies used

Primary antibodies:
mouse anti-γ-tubulin (GTU88, T5326, lot number:0000216733, Sigma-Aldrich) used at 1:500.
Secondary antibodies:
Goat Anti-Mouse IgG H&L (Alexa Fluor® 555, ab150118, lot number:1062774-2, abcam) used at 1:500.

Validation

All antibodies were previously validated by published work. Relevant studies include:
γ-tubulin:Specific staining of spindle poles in human oocytes, mouse oocytes and human somatic cell (Holubcová, Z., et al. 2015 Science 348(6239): 1143-1147).

Plants

Seed stocks

Report on the source of all seed stocks or other plant material used. If applicable, state the seed stock centre and catalogue number. If plant specimens were collected from the field, describe the collection location, date and sampling procedures.

Novel plant genotypes

Describe the methods by which all novel plant genotypes were produced. This includes those generated by transgenic approaches, gene editing, chemical/radiation-based mutagenesis and hybridization. For transgenic lines, describe the transformation method, the number of independent lines analyzed and the generation upon which experiments were performed. For gene-edited lines, describe the editor used, the endogenous sequence targeted for editing, the targeting guide RNA sequence (if applicable) and how the editor was applied.

Authentication

Describe any authentication procedures for each seed stock used or novel genotype generated. Describe any experiments used to assess the effect of a mutation and, where applicable, how potential secondary effects (e.g. second site T-DNA insertions, mosiacism, off-target gene editing) were examined.
